# Anatomical Proximity of Posterior Maxillary Tooth Roots as an Independent Predictor of Maxillary Sinus Mucosal Thickening: A CBCT-Based Study

**DOI:** 10.3390/diagnostics16162675

**Published:** 2026-08-21

**Authors:** Cemile Nur Yıldırım, Ali Altındağ

**Affiliations:** 1 Dentomaxillofacial Radiology, Faculty of Dentistry, İstanbul Aydın University, Istanbul 34295, Türkiye; yildirimcemilenur@gmail.com; 2 Dentomaxillofacial Radiology, Faculty of Dentistry, Necmettin Erbakan University, Konya 42090, Türkiye

**Keywords:** cone-beam computed tomography, maxillary sinus, molars, mucosal thickening

## Abstract

**Objectives:** This study aimed to evaluate the association of anatomical proximity between posterior maxillary tooth roots and the sinus floor with the presence and severity of maxillary sinus mucosal thickening (MT) using cone-beam computed tomography (CBCT). **Materials and Methods:** A retrospective CBCT-based analysis was performed on 300 patients (175 females, 125 males; mean age: 27.55 ± 8.74 years). Root–sinus anatomical relationships were classified into three proximity types based on increasing root–sinus closeness. Maxillary sinus mucosal thickness was measured in millimeters, and mucosal thickening was defined using both a binary threshold (>2 mm vs. ≤2 mm) and an ordinal severity classification (≤2 mm, 2–10 mm, >10 mm). Multivariable binary logistic regression models evaluated the presence of MT after adjustment for age, sex, diabetes, cardiovascular disease, and smoking. As the proportional odds assumption was not satisfied, MT severity was evaluated using multinomial logistic regression models adjusted for age and sex. **Results:** Mucosal thickening (>2 mm) was observed in 34.7% of patients on the right side and 34.7% on the left side, with bilateral involvement in 19.7%. Proximity type was significantly associated with mucosal thickening on the right and left sides (*p* < 0.01). In multivariable binary logistic regression, each one-unit increase in proximity type was associated with a 2.58-fold increase in the odds of mucosal thickening on the right side (OR = 2.58; 95% CI: 1.61–4.12; *p* < 0.001) and a 1.93-fold increase on the left side (OR = 1.93; 95% CI: 1.23–3.03; *p* = 0.004). In age- and sex-adjusted multinomial logistic regression, each one-unit increase in proximity type was associated with moderate mucosal thickening on both the right (OR = 2.60; 95% CI: 1.56–4.32; *p* < 0.001) and left sides (OR = 1.74; 95% CI: 1.06–2.87; *p* = 0.030), relative to normal mucosal thickness. Proximity type was also associated with severe mucosal thickening on the right (OR = 2.75; 95% CI: 1.26–6.04; *p* = 0.012) and left sides (OR = 2.79; 95% CI: 1.34–5.84; *p* = 0.006). Age showed a modest independent association in the binary models, whereas systemic factors, including diabetes, cardiovascular disease, and smoking, were not significantly associated with mucosal thickening. Sensitivity analyses using an alternative severity classification supported the association between proximity type and severe mucosal thickening on both sides, although the association with moderate left-side mucosal thickening was threshold-dependent. **Conclusions:** Anatomical proximity between posterior maxillary tooth roots and the sinus floor was independently associated with MT presence after adjustment for demographic and systemic factors and with MT severity after adjustment for age and sex. These findings support considering root–sinus proximity alongside odontogenic and sinonasal factors during CBCT-based assessment and treatment planning in the posterior maxilla.

## 1. Introduction

The maxillary sinus is the largest paranasal sinus and demonstrates close anatomical proximity to the posterior maxillary dentition, resulting in a complex structural and functional relationship between dental and sinonasal tissues [1,2]. The Schneiderian membrane lining the maxillary sinus is a biologically active mucosa that may undergo adaptive or pathological thickening in response to inflammatory, infectious, or mechanical stimuli. Radiographically, maxillary sinus mucosal thickening (MT) represents one of the most frequently observed incidental findings in dental imaging and may reflect inflammatory or other mucosal responses, although it is not, by itself, diagnostic of clinical sinusitis [3,4].

Cone-beam computed tomography (CBCT) is widely used when three-dimensional evaluation of maxillary sinus anatomy is clinically indicated because of its high spatial resolution and multiplanar visualization capabilities [5]. Compared with conventional two-dimensional radiography, CBCT allows direct measurement of mucosal thickness and three-dimensional assessment of the anatomical relationship between posterior maxillary tooth roots and the sinus floor [6,7,8]. This three-dimensional assessment can help characterize anatomical relationships relevant to radiographic interpretation and preoperative evaluation, particularly before implant-related procedures in the posterior maxilla [9].

The reported prevalence of MT in CBCT-based studies ranges from 30% to 60%, even in asymptomatic populations [10,11,12]. Although a universally accepted pathological threshold has not been established, mucosal thickness exceeding 2–3 mm is commonly considered indicative of abnormal mucosal alteration [3,10,13,14]. Previous studies have demonstrated significant associations between odontogenic pathologies—such as periapical lesions, periodontal bone loss, and endodontic infections—and MT, supporting a potential odontogenic contribution to maxillary sinus mucosal changes [15,16,17].

In addition to overt dental disease, anatomical factors have been proposed as potential contributors to sinus mucosal changes. In particular, reduced bony separation between posterior maxillary tooth roots and the sinus floor has been hypothesized to influence the relationship between odontogenic conditions and sinus mucosal changes; however, anatomical proximity alone does not establish inflammatory transmission or an odontogenic origin. While healthy, non-carious teeth are unlikely to represent overt inflammatory sources, subtle endodontic or periodontal involvement may remain clinically silent and undetectable on CBCT, with close root–sinus proximity potentially facilitating inflammatory spread to the Schneiderian membrane. However, existing evidence regarding the independent role of anatomical proximity remains inconclusive. Aguori et al. [18] evaluated healthy posterior teeth and found no significant association between tooth–sinus proximity and MT. Other CBCT studies have demonstrated substantial anatomical variability in root–sinus relationships and frequent root contact with or protrusion into the sinus cavity, particularly in molar regions [19,20,21]. Although many previous investigations have primarily used descriptive or univariable approaches, more recent studies have begun to apply multivariable and multilevel analyses when evaluating odontogenic risk factors [22], leaving it unclear whether anatomical proximity is independently associated with both the occurrence and severity of MT in multivariable analyses.

From a clinical perspective, clarifying this association may contribute to the interpretation of CBCT findings and to a more contextual assessment of the posterior maxilla, although any potential clinical implications must be considered alongside odontogenic and sinonasal factors. Therefore, this study aimed to evaluate whether the anatomical proximity of posterior maxillary tooth roots to the sinus floor was associated with the presence and severity of maxillary sinus mucosal thickening using CBCT, with multivariable adjustment appropriate to each outcome.

## 2. Methods

### 2.1. Study Design and Sample

This retrospective cross-sectional study was conducted using cone-beam computed tomography (CBCT) images obtained from patients who attended the Department of Oral and Maxillofacial Radiology for routine diagnostic purposes. CBCT examinations had been originally performed for various clinical indications, including implant planning, assessment of impacted teeth, and evaluation of dentomaxillofacial structures. The institutional CBCT archive was retrospectively screened for examinations acquired between January 2021 and December 2024. After application of the predefined eligibility criteria, eligible examinations were selected using a computer-generated random sampling procedure. The study protocol was approved by the Local Institutional Review Board (Approval No:2024/526—Approval Date: 26 December 2024), and the study was conducted in accordance with the Declaration of Helsinki. Clinical trial registration was not applicable.

An a priori power analysis was performed using G*Power version 3.1.9.7 (Heinrich-Heine-Universität Düsseldorf, Düsseldorf, Germany) to determine the minimum required sample size. The analysis was based on the chi-square test of independence, which was considered a conservative approximation for the primary association between proximity type and mucosal thickening (>2 mm vs. ≤2 mm). Parameters were defined as follows: medium effect size (w = 0.30), significance level (α = 0.05), statistical power (1−β = 0.95), and degrees of freedom = 2. The analysis indicated that a minimum of 172 subjects was required. The final sample size of 300 patients exceeded this minimum requirement.

A total of 300 patients were included in the study. Inclusion criteria were:

(1) availability of high-quality CBCT scans including both maxillary sinuses and posterior maxillary teeth,

(2) absence of obvious maxillary sinus pathology such as cysts, tumors, or previous sinus surgery,

(3) presence of at least one posterior maxillary tooth whose root apex was located beneath or within the anatomical boundaries of the maxillary sinus floor on multiplanar CBCT images. No predefined distance cut-off was applied because root apices without sinus-floor contact were included as Type 1 relationships.

Exclusion criteria included poor image quality, motion artifacts, history of maxillary sinus surgery, and conditions that could obscure accurate evaluation of the sinus mucosa or tooth–sinus relationship. To minimize potential confounding from odontogenic sources of inflammation, scans were also excluded when the posterior maxillary teeth adjacent to the sinus floor exhibited periapical radiolucency, previous root canal treatment, extensive caries approaching the pulp, or advanced periodontal bone loss. Clinical pulp-vitality testing was unavailable because of the retrospective imaging-based design; therefore, odontogenic screening was based on radiographically detectable findings. These conditions were assessed on multiplanar CBCT reconstructions during the initial screening process.

### 2.2. CBCT İmage Acquisition and Evaluation

All CBCT examinations were performed using a 3D Accuitomo 170 system (J Morita Mfg. Corp., Kyoto, Japan), operating at 90 kVp and 5 mA with exposure times ranging from 15 to 18 s, in accordance with the manufacturer’s recommended imaging protocol. As this was a retrospective study, the examinations had originally been acquired for various clinical indications rather than specifically for the present research. Only examinations acquired with a field of view of 170 × 120 mm and a voxel size of 0.25 mm that fully included both maxillary sinuses and the posterior maxillary teeth were selected for analysis.

All images were evaluated using dedicated CBCT viewing software (i-Dixel, J. Morita Mfg. Corp., Kyoto, Japan) under standardized viewing conditions, including controlled ambient lighting and calibrated display monitors. Multiplanar reconstruction (MPR) images were analyzed in axial, coronal, and sagittal planes to ensure accurate anatomical assessment. The coronal plane was primarily used for evaluating the anatomical relationship between posterior tooth roots and the sinus floor and for measuring mucosal thickness.

Before the formal image assessment, the two observers participated in a calibration session in which the evaluation criteria, proximity-type definitions, and mucosal-thickness measurement procedure were reviewed using representative CBCT scans that were not included in the final study sample. During the formal assessment, each observer independently assessed the side-specific proximity type, maximum mucosal thickness in millimeters, and the presence of radiographically detectable odontogenic exclusion criteria.

### 2.3. Assessment of Proximity Type

**Figure 1:**
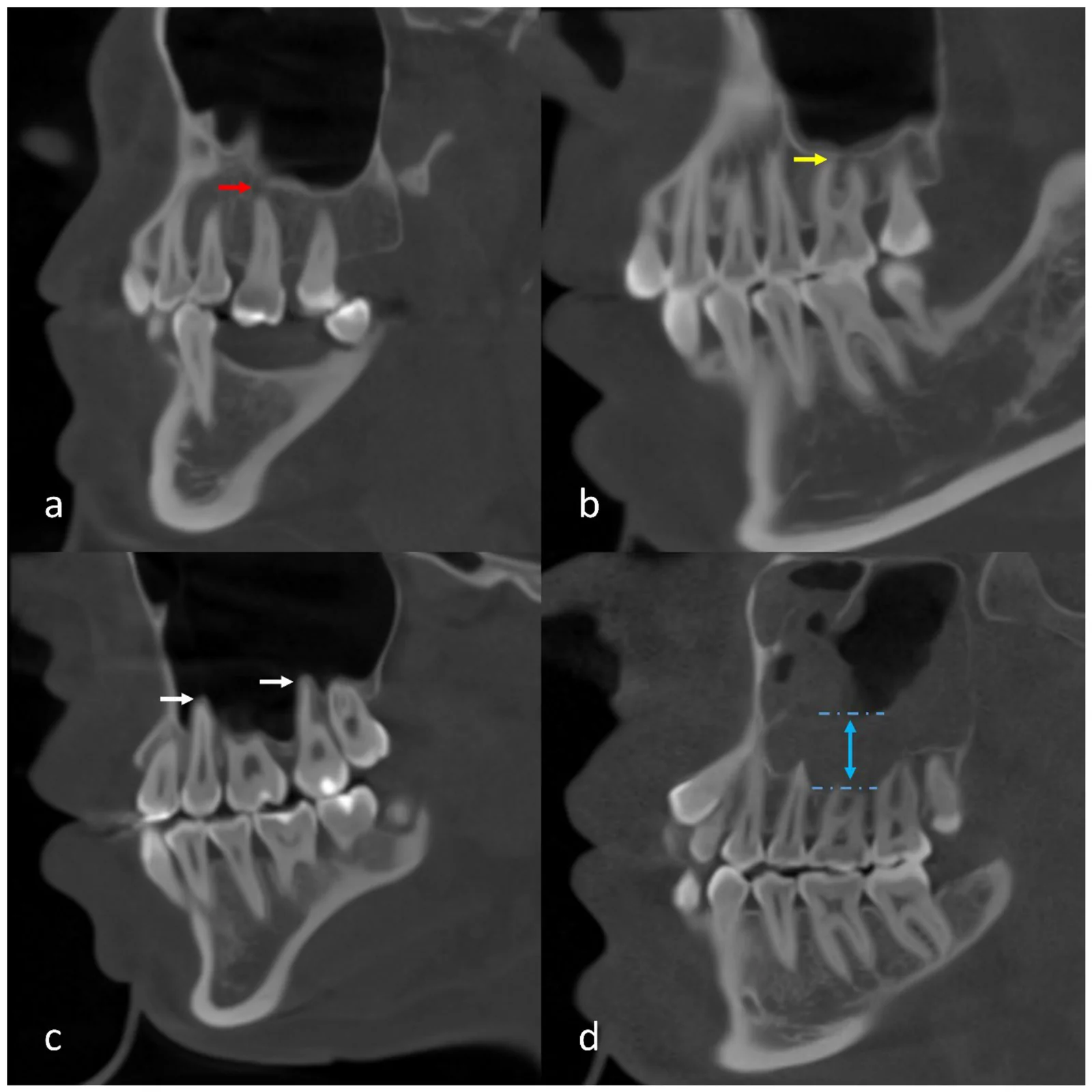
Representative CBCT images demonstrating the classification of anatomical proximity between maxillary posterior tooth roots and the sinus floor, along with mucosal thickening (MT) measurements. (**a**) Type 1: The root apex (red arrow) is located below the maxillary sinus floor without contact. (**b**) Type 2: The root apex is in direct contact with the cortical boundary of the sinus floor (yellow arrow). (**c**) Type 3: The root apices protrude into the maxillary sinus cavity (white arrows). (**d**) Severe mucosal thickening in a Type 3 relationship: mucosal thickening exceeding 10 mm, with the blue double-headed arrow indicating the measurement.

The anatomical relationship between the roots of posterior maxillary teeth and the maxillary sinus floor was classified into three proximity types based on their spatial relationship [19,20,21] (Figure 1):

- Type 1: Root apex located below the sinus floor without contact.
- Type 2: Root apex in direct contact with the sinus floor.
- Type 3: Root apex protruding into the maxillary sinus cavity.

Proximity type was assessed separately for the right and left sides. When multiple posterior teeth were present, the tooth exhibiting the closest anatomical relationship to the sinus floor was used for classification. This approach was adopted to represent the highest anatomical risk for each maxillary sinus and to provide a single clinically relevant proximity category for statistical analysis.

### 2.4. Measurement of Maxillary Sinus Mucosal Thickening

Maxillary sinus mucosal thickness was measured as the maximum perpendicular distance from the sinus wall to the mucosal surface at the thickest location. Measurements were recorded in millimeters for both maxillary sinuses.

Mucosal thickening was defined based on established radiological thresholds and categorized using binary and severity classifications.

1. Primary severity classification (main model):
  - Normal: ≤2 mm
  - Moderate: >2–10 mm
  - Severe: >10 mm
2. Alternative severity classification (sensitivity analysis):
  - Normal: ≤2 mm
  - Moderate: >2–8 mm
  - Severe: >8 mm

For binary analyses, mucosal thickening was dichotomized as ≤2 mm and >2 mm.

### 2.5. Observer Reliability

Proximity-type classification was performed independently by two experienced oral and maxillofacial radiologists (CNY and AA), who were blinded to each other’s assessments. Inter-observer agreement for proximity-type classification was evaluated by comparing the independent assessments of the two observers across all 300 CBCT scans. Disagreements in proximity-type classification were jointly re-evaluated and resolved by consensus, and the consensus classifications were used in the statistical analyses.

All mucosal-thickness measurements were performed by CNY. To assess intra-observer reliability, CNY repeated both the proximity-type classification and mucosal-thickness measurements in a randomly selected subset of 30 CBCT scans (10% of the study sample) after a three-week interval. Cohen’s kappa coefficient was used to assess inter-observer agreement and intra-observer agreement for proximity-type classification, whereas the intraclass correlation coefficient (ICC) was used to assess the intra-observer reproducibility of CNY’s mucosal-thickness measurements.

### 2.6. Statistical Analysis

Statistical analyses were performed using IBM SPSS Statistics for Windows, Version 27.0 (IBM Corp., Armonk, NY, USA). Descriptive statistics were calculated for demographic characteristics, proximity types, and mucosal thickening (MT).

Associations between proximity type and binary MT (>2 mm) were initially assessed using the chi-square test, while differences in continuous MT values among proximity types were evaluated using the Kruskal–Wallis test.

Multivariable binary logistic regression models were constructed to evaluate the association between proximity type and the presence of MT (>2 mm) after adjustment for age, sex, diabetes, cardiovascular disease, and smoking. Separate models were fitted for the right and left sides. The proportional odds assumption for ordinal logistic regression was evaluated using the test of parallel lines. Because this assumption was not satisfied, multinomial logistic regression was used to evaluate MT severity, with normal MT (≤2 mm) as the reference outcome. The primary multinomial models compared moderate (>2–10 mm) and severe (>10 mm) MT with normal MT. Because diabetes, cardiovascular disease, and smoking produced zero or very small cell counts across the severity categories, they were excluded from the multinomial models, which were adjusted for proximity type, age, and sex.

Sensitivity analyses were performed using an alternative severity classification (≤2 mm, 2–8 mm, >8 mm) to evaluate the robustness of the findings. Odds ratios (ORs) with 95% confidence intervals (CIs) were reported, and statistical significance was set at *p* < 0.05.

## 3. Results

Inter-observer agreement between CNY and AA for proximity-type classification was excellent (Cohen’s κ = 0.97). In the subset of 30 CBCT scans that were reassessed after a three-week interval, intra-observer agreement for CNY’s proximity-type classifications was also excellent (Cohen’s κ = 0.99). The intra-observer reproducibility of CNY’s mucosal-thickness measurements was excellent, with an ICC of 0.95 (95% CI: 0.92–0.97).

A total of 300 patients were included. The mean age was 27.55 ± 8.74 years (range: 20–60 years). Of the participants, 175 (58.3%) were female and 125 (41.7%) were male (Table 1). The prevalence of diabetes, cardiovascular disease, and smoking was 4.3%, 3.0%, and 5.7%, respectively.

### 3.1. Distribution of Proximity Types and Prevalence of Mucosal Thickening

Proximity Type 2 was the most frequently observed anatomical pattern on both sides. On the right side, Type 2 was observed in 187 patients (62.3%), followed by Type 3 in 88 patients (29.3%) and Type 1 in 25 patients (8.3%). Similarly, on the left side, Type 2 was present in 177 patients (59.0%), followed by Type 3 in 96 patients (32.0%) and Type 1 in 27 patients (9.0%) (Table 2).

Mucosal thickening (>2 mm) was observed in 104 patients (34.7%) on the right side and 104 patients (34.7%) on the left side. Bilateral mucosal thickening was identified in 59 patients (19.7%), whereas unilateral mucosal thickening was observed in 90 patients (30.0%). Among patients with unilateral involvement, 45 had right-sided and 45 had left-sided mucosal thickening.

### 3.2. Association Between Proximity Type and Mucosal Thickening

A statistically significant association was observed between proximity type and the presence of maxillary sinus mucosal thickening greater than 2 mm on both sides (Table 3). On the right side, the prevalence of mucosal thickening increased progressively from Type 1 (20.0%) to Type 2 (28.9%) and Type 3 (51.1%) (χ^2^ = 15.68, *p* < 0.001).

Similarly, on the left side, mucosal thickening was significantly more frequent in Type 3 proximity (46.9%) compared to Type 2 (29.4%) and Type 1 (25.9%) (χ^2^ = 9.41, *p* = 0.009).

When mucosal thickening was analyzed as a continuous variable, significant differences were also detected among proximity types on both sides. Kruskal–Wallis analysis demonstrated a statistically significant difference in MT across proximity groups on the right side (H = 15.63, *p* < 0.001) and on the left side (H = 10.53, *p* = 0.005).

### 3.3. Multivariable Binary Prediction of Mucosal Thickening

In multivariable logistic regression analysis, proximity type remained a strong and independent predictor of maxillary sinus mucosal thickening greater than 2 mm on both sides after adjustment for age, sex, diabetes, cardiovascular disease, and smoking status (Table 4). On the right side, each one-unit increase in proximity type was associated with a 2.58-fold increase in the odds of mucosal thickening (OR = 2.58; 95% CI: 1.61–4.12; *p* < 0.001). Similarly, on the left side, proximity type also demonstrated a significant association with mucosal thickening (OR = 1.93; 95% CI: 1.23–3.03; *p* = 0.004).

Age was also independently associated with mucosal thickening on both sides, with OR values of 1.05 (95% CI: 1.01–1.08; *p* = 0.007) on the right side and 1.04 (95% CI: 1.01–1.08; *p* = 0.018) on the left side.

Sex was not a significant predictor in the right-side model (OR = 1.14; *p* = 0.607). However, male sex was significantly associated with increased odds of mucosal thickening on the left side, with males exhibiting nearly 1.9 times higher odds compared with females (OR = 1.85; 95% CI: 1.12–3.07; *p* = 0.017).

Diabetes, cardiovascular disease, and smoking were not significantly associated with mucosal thickening in the adjusted models (all *p* > 0.05).

### 3.4. Multinomial Analysis of Mucosal-Thickening Severity

The test of parallel lines indicated that the proportional odds assumption was not satisfied for either the right-side model (χ^2^(6) = 13.44, *p* = 0.037) or the left-side model (χ^2^(6) = 15.53, *p* = 0.016). Therefore, multinomial logistic regression was used to evaluate MT severity, with normal MT (≤2 mm) as the reference category. The primary classification comprised 196 normal, 78 moderate, and 26 severe observations on the right side and 196 normal, 74 moderate, and 30 severe observations on the left side.

Both multinomial models were statistically significant overall (right: likelihood-ratio χ^2^(6) = 23.24, *p* < 0.001; left: likelihood-ratio χ^2^(6) = 30.38, *p* < 0.001). On the right side, each one-unit increase in proximity type was associated with 2.60-fold higher odds of moderate MT (OR = 2.60; 95% CI: 1.56–4.32; *p* < 0.001) and 2.75-fold higher odds of severe MT (OR = 2.75; 95% CI: 1.26–6.04; *p* = 0.012), relative to normal MT. Age was positively associated with moderate MT (OR = 1.04; 95% CI: 1.01–1.07; *p* = 0.019) but not with severe MT (*p* = 0.249). Sex was not significantly associated with either outcome category (Table 5).

On the left side, each one-unit increase in proximity type was associated with 1.74-fold higher odds of moderate MT (OR = 1.74; 95% CI: 1.06–2.87; *p* = 0.030) and 2.79-fold higher odds of severe MT (OR = 2.79; 95% CI: 1.34–5.84; *p* = 0.006), relative to normal MT. Age was associated with moderate MT (OR = 1.05; 95% CI: 1.02–1.08; *p* = 0.002) but not with severe MT (*p* = 0.537). Male sex showed borderline associations with moderate and severe MT, but neither reached statistical significance.

In the sensitivity analysis using the alternative thresholds, proximity type remained associated with severe MT on both sides (right: OR = 2.93; 95% CI: 1.44–5.97; *p* = 0.003; left: OR = 3.26; 95% CI: 1.69–6.28; *p* < 0.001). It was also associated with moderate MT on the right side (OR = 2.51; 95% CI: 1.48–4.24; *p* < 0.001), whereas the association with moderate MT on the left side was not statistically significant (OR = 1.46; 95% CI: 0.86–2.47; *p* = 0.158). Thus, the association between proximity type and severe MT was consistent across both severity classifications, while the association with moderate left-side MT was sensitive to the selected threshold.

## 4. Discussion

Maxillary sinus mucosal thickening (MT) is a commonly reported radiographic finding on cone-beam computed tomography (CBCT) examinations and has been associated with odontogenic conditions such as periapical lesions, periodontal disease, and other inflammatory dental conditions. Previous observational studies and systematic reviews have reported associations between dental pathologies and maxillary sinus mucosal thickening, with periodontal bone loss and periapical lesions being among the most consistently reported associated factors [3,23,24]. Meta-analytic evidence has similarly shown that odontogenic lesions are associated with a higher occurrence of sinus mucosal thickening and odontogenic maxillary sinusitis, although the observational nature of the available evidence limits causal interpretation [23,25].

Although dental pathology is an established contributor to maxillary sinus changes, the role of anatomical proximity remains incompletely understood. The present study extends previous CBCT research [24,26] by demonstrating an association between root–sinus proximity and MT presence after adjustment for demographic and systemic factors, as well as with moderate and severe MT after adjustment for age and sex. These findings support anatomical proximity as one structural correlate within a multifactorial process, without establishing causality or an odontogenic origin.

The present study was conducted in a relatively young cohort with a mean age of 27.55 ± 8.74 years and a female predominance, which should be taken into account when interpreting the findings. Nevertheless, this demographic profile is comparable to several CBCT-based studies investigating incidental maxillary sinus findings in dental populations, where young and middle-aged adults constitute the majority of examined subjects [27,28]. These studies indicate that incidental sinus alterations can also be detected in younger individuals, although some evidence suggests that the prevalence and extent of mucosal changes may increase with age.

Consistent with this possibility, Hsiao et al. [29] reported a significantly higher prevalence of maxillary sinus mucosal thickening in older age groups, suggesting a possible association between age and Schneiderian membrane changes. In the present study, age showed modest associations with binary MT on both sides and with moderate, but not severe, MT in the multinomial models. These findings suggest that age may contribute to MT but is unlikely to explain the observed variation on its own. Local anatomical factors and tooth–sinus relationships may instead contribute alongside demographic, odontogenic, and sinonasal factors to the mucosal response, which is consistent with previous CBCT-based investigations emphasizing the multifactorial nature of sinus mucosal changes [30]. Gu et al. [31] further reported that the distance between posterior root apices and the sinus floor increased with age, whereas root–sinus proximity patterns showed relatively limited variation according to sex and side. These observations provide additional anatomical context but do not establish root–sinus relationships as stable determinants of MT across demographic groups.

The observed increase in MT frequency from Type 1 to Type 3 is consistent with a graded association between root–sinus proximity and MT. Reduced bony separation has been proposed as a factor that may facilitate a local mucosal response [26,32]; however, proximity alone does not establish the cause, odontogenic origin, or clinical significance of MT.

In line with these findings, Gu et al. [31] reported that root apices extending into or contacting the maxillary sinus (Type IS and Type CO) were most frequently observed in palatal roots of first molars and mesiobuccal roots of second molars, anatomical sites that are also commonly implicated in odontogenic sinus pathology. Similarly, Ok et al. [33] reported that maxillary molars, particularly second molars, had the closest anatomical relationships with the sinus floor in a Turkish population. Together, these studies illustrate the recurring involvement of molar roots in close root–sinus relationships across different populations, although they do not directly establish an association with MT.

Nair et al. [32] proposed that minimal bony separation between root apices and the sinus floor may reduce the anatomical barrier to inflammatory diffusion, thereby increasing the susceptibility of the Schneiderian membrane to thickening. Likewise, Yusufoğlu et al. [26] reported associations between variations in tooth–sinus relationships and sinus mucosal thickening, particularly when periapical or periodontal pathology was present. Taken together, these findings provide biological context for the association observed in the present study. However, because radiographically detectable dental pathology was excluded and clinical pulp-vitality and detailed periodontal data were unavailable, the present findings cannot establish the underlying mechanism or confirm an odontogenic source of MT [15,34].

From a surgical and anatomical perspective, additional context is provided by the three-dimensional CBCT reconstruction study of Huang et al. [35], who reported that the palatal roots of maxillary molars frequently had root-apex planes located at or above the sinus floor, a relationship relevant to planning endodontic microsurgery. Although Huang et al. [35] focused primarily on surgical access pathways, their morphometric data illustrate the limited anatomical separation that may occur between molar roots and the maxillary sinus. Nevertheless, these anatomical findings should not be interpreted as direct evidence of increased mucosal reactivity or pathological sinus involvement.

A key strength of this study is the separate evaluation of MT presence and severity using binary and multinomial logistic regression. As the proportional odds assumption was not satisfied, severity was evaluated using age- and sex-adjusted multinomial models. The associations between proximity type and severe MT remained statistically significant under both severity classifications, whereas the association with moderate left-side MT varied according to the threshold applied. This threshold dependence warrants caution when interpreting associations involving moderate MT.

Whereas Jouhar et al. [36] and Kaimal and Patil [37] emphasized the association of periapical and periodontal pathologies with MT, the present findings indicate that anatomical proximity may also be relevant in the absence of radiographically detectable dental disease. The multivariable approach used in this study extends the predominantly descriptive or univariable findings reported by Gu et al. [31] and Ok et al. [33].

Interestingly, male sex emerged as a significant predictor of mucosal thickening on the left side but not on the right. Although the clinical relevance of this asymmetry remains unclear, similar sex-related tendencies have been reported in previous studies [29,36]. This finding may reflect subtle differences in craniofacial morphology or sinus anatomy between sexes, as suggested in morphometric CBCT studies [19,21]. Gu et al. [31], however, reported minimal sex-related differences in root–sinus distance, suggesting that sex effects may be context-dependent and potentially modulated by population-specific anatomical characteristics.

The use of a 2 mm threshold to define pathological mucosal thickening in the present study is consistent with widely accepted radiological criteria in the literature [38,39]. Terlemez et al. [39] reported substantial variability in the prevalence of mucosal thickening, ranging from 12% to over 60%, depending on population characteristics, imaging protocols, and diagnostic thresholds. This variability highlights the importance of standardized definitions when comparing results across studies.

Diabetes, cardiovascular disease, and smoking were not statistically significantly associated with mucosal thickening in the adjusted models. This finding is broadly consistent with earlier CBCT-based studies reporting limited or inconsistent associations between systemic variables and sinus pathology [23,38,40]. However, these conditions occurred at relatively low frequencies in the present sample (4.3%, 3.0%, and 5.7%, respectively), resulting in imprecise estimates and wide confidence intervals. This was particularly evident for cardiovascular disease in the right-side binary model (OR = 0.13; 95% CI: 0.01–1.28). Therefore, the absence of statistical significance should not be interpreted as evidence that these systemic factors have no association with mucosal thickening or that local factors necessarily have a more dominant role.

From a clinical perspective, evaluation of the root–sinus relationship may complement the assessment of other dental and sinonasal findings when CBCT has been obtained for an appropriate clinical indication. Type 3 proximity, in which the root apex protrudes into the maxillary sinus, may warrant particular attention before implant placement, sinus floor elevation, endodontic microsurgery, or other procedures involving the posterior maxilla. However, the presence of Type 3 proximity or MT alone should not be regarded as evidence of sinus disease or as an indication for additional imaging or treatment.

Bilateral MT was present in 19.7% of patients and may reflect generalized sinonasal, inflammatory, allergic, or environmental influences rather than a solely localized odontogenic mechanism. Therefore, the side-specific associations observed in this study should not be interpreted as establishing a dental origin, particularly because clinical sinonasal and otorhinolaryngological data were unavailable.

### Limitations

This study has several limitations. Its retrospective design precludes causal inference. In addition, the use of a single-center dental CBCT archive may have introduced referral-related selection bias and may limit the generalizability of the findings to broader populations. The CBCT examinations had also been acquired for heterogeneous clinical indications, including implant planning, impacted-tooth assessment, and evaluation of dentomaxillofacial structures, which may have contributed to variability in the characteristics of the study sample. In the absence of otorhinolaryngological examination, sinonasal symptom assessment, nasal endoscopy, or a clinical diagnosis of sinusitis, MT should be interpreted exclusively as a radiographic finding and not as clinically significant sinusitis. Detailed periodontal parameters beyond the predefined screening criteria and sinonasal clinical history were not systematically evaluated. Furthermore, clinical pulp-vitality data were unavailable because of the retrospective imaging-based design. Consequently, although teeth with radiographically detectable odontogenic pathology were excluded, clinically silent pulpal or periapical conditions without evident CBCT findings could not be completely ruled out. Moreover, the low frequencies of diabetes, cardiovascular disease, and smoking produced imprecise estimates in the binary models and sparse cells in the multinomial categories. These variables were therefore excluded from the severity models, which were adjusted only for age and sex, leaving the possibility of residual confounding. Side-level classification was based on the tooth with the closest relationship to the sinus floor. Because the selected tooth and root were not recorded separately, tooth- or root-specific distributions and clustered analyses using GEE or multilevel models could not be performed. Future studies should retain all tooth- and root-level observations and account for within-patient clustering.

## 5. Conclusions

Closer root–sinus proximity was independently associated with the presence of MT, with each one-unit increase in proximity type increasing the odds by 2.58-fold on the right (95% CI: 1.61–4.12) and 1.93-fold on the left (95% CI: 1.23–3.03) after adjustment for demographic and systemic factors. It was also associated with moderate and severe MT after adjustment for age and sex. Anatomical proximity should be considered one radiographic correlate within the multifactorial nature of MT rather than its sole determinant. When CBCT is obtained for an appropriate clinical indication, assessment of root–sinus proximity may complement odontogenic and sinonasal evaluation during treatment planning in the posterior maxilla.

## Figures and Tables

**Table 1:** Demographic characteristics of the study population.

Gender	n	Mean Age (Years) ± SD	Min	Max
Female	175	26.84 ± 7.82	20	58
Male	125	28.54 ± 9.84	20	60
Total	300	27.55 ± 8.74	20	60

**Table 2:** Distribution of proximity types and prevalence of mucosal thickening (>2 mm).

Side	Type 1 n (%)	Type 2 n (%)	Type 3 n (%)	MT > 2 mm n (%)
**Right**	25 (8.3%)	187 (62.3%)	88 (29.3%)	104 (34.7%)
**Left**	27 (9.0%)	177 (59.0%)	96 (32.0%)	104 (34.7%)

**Table 3:** The distribution of maxillary sinus mucosal thickening (MT) greater than 2 mm according to proximity type for the right and left sides.

Proximity Type	Right Side MT ≤ 2 mm n (%)	Right Side MT > 2 mm n (%)	Left Side MT ≤ 2 mm n (%)	Left Side MT > 2 mm n (%)
Type 1	20 (80.0%)	5 (20.0%)	20 (74.1%)	7 (25.9%)
Type 2	133 (71.1%)	54 (28.9%)	125 (70.6%)	52 (29.4%)
Type 3	43 (48.9%)	45 (51.1%)	51 (53.1%)	45 (46.9%)
**Chi-square (*p*)**	-	**15.68 (<0.001)**	-	**9.41 (0.009)**

**Table 4:** Multivariable binary logistic regression for mucosal thickening (>2 mm).

Variable	Right OR (95% CI)	*p* (Right)	Left OR (95% CI)	*p* (Left)
**Proximity type (per unit)**	2.58 (1.61–4.12)	<0.001	1.93 (1.23–3.03)	0.004
**Age (per year)**	1.05 (1.01–1.08)	0.007	1.04 (1.01–1.08)	0.018
**Sex (male)**	1.14 (0.68–1.91)	0.607	1.85 (1.12–3.07)	0.017
**Diabetes**	0.98 (0.25–3.85)	0.979	1.86 (0.49–6.97)	0.359
**CVD**	0.13 (0.01–1.28)	0.081	0.22 (0.03–1.42)	0.111
**Smoking**	1.57 (0.52–4.80)	0.426	1.57 (0.53–4.66)	0.419

**Table 5:** Multinomial logistic regression for mucosal-thickening severity (primary classification: ≤2 mm, >2–10 mm, and >10 mm).

Outcome Comparison	Variable	Right OR (95% CI)	*p* (Right)	Left OR (95% CI)	*p* (Left)
Moderate vs. normal	Proximity type (per unit)	2.60 (1.56–4.32)	<0.001	1.74 (1.06–2.87)	0.030
	Age (per year)	1.04 (1.01–1.07)	0.019	1.05 (1.02–1.08)	0.002
	Sex (male)	0.99 (0.57–1.74)	0.981	1.74 (1.00–3.05)	0.052
Severe vs. normal	Proximity type (per unit)	2.75 (1.26–6.04)	0.012	2.79 (1.34–5.84)	0.006
	Age (per year)	1.03 (0.98–1.08)	0.249	0.98 (0.92–1.04)	0.537
	Sex (male)	1.77 (0.76–4.16)	0.188	2.13 (0.96–4.73)	0.063

Normal MT (≤2 mm) was used as the reference outcome. Female sex was the reference category. The models were mutually adjusted for proximity type, age, and sex. Overall model fit was significant for both the right-side model (likelihood-ratio χ^2^(6) = 23.24, *p* < 0.001) and the left-side model (likelihood-ratio χ^2^(6) = 30.38, *p* < 0.001).

## Data Availability

The data presented in this study are available from the corresponding author upon reasonable request. The data are not publicly available due to patient privacy and ethical restrictions.
